# Novel Hierarchical Fe(III)-Doped Cu-MOFs With Enhanced Adsorption of Benzene Vapor

**DOI:** 10.3389/fchem.2019.00652

**Published:** 2019-09-27

**Authors:** Xuejiao Sun, Xiulian Gu, Wentao Xu, Wen-Jie Chen, Qibin Xia, Xiaoyang Pan, Xiaojing Zhao, Yi Li, Qi-Hui Wu

**Affiliations:** ^1^School of Chemical Engineering and Materials Science, Quanzhou Normal University, Quanzhou, China; ^2^School of Chemistry and Chemical Engineering, South China University of Technology, Guangzhou, China; ^3^Jiangsu Key Laboratory of Advanced Functional Polymer Designand Application, Department of Polymer Science and Engineering, College of Chemistry, Chemical Engineering and Materials Science, Soochow University, Suzhou, China; ^4^College of Mechanical and Energy Engineering, Jimei University, Xiamen, China

**Keywords:** HKUST-1, hierarchical-pore, adsorption, benzene, VOC

## Abstract

New hierarchical Fe(III)-doped Cu-MOFs (Fe-HK) were developed via introduction of Fe^3+^ ions during HKUST-1 synthesis. The obtained products were characterized by N_2_ adsorption, X-ray diffraction, scanning electron microscopy, energy dispersive spectroscopy, FTIR spectroscopy, and thermal analysis. The adsorption isotherms and kinetics of benzene vapor were measured and consecutive adsorption–desorption cycles were performed. It was found that the hierarchical-pore Fe-HK-2 exhibited optimal textural properties with high BET surface area of 1,707 m^2^/g and total pore volume of 0.93 cm^3^/g, which were higher than those of the unmodified HKUST-1. Significantly, the hierarchical-pore Fe-HK-2 possessed outstanding benzene adsorption capacity, which was 1.5 times greater than the value on HKUST-1. Benzene diffusivity of Fe-HK-2 was 1.7 times faster than that of parent HKUST-1. Furthermore, the benzene adsorption on Fe-HK-2 was highly reversible. The hierarchical-pore Fe-HK-2 with high porosity, outstanding adsorption capacity, enhanced diffusion rate, and excellent reversibility might be an attractive candidate for VOCs adsorption. This may offer a simple and effective strategy to synthesize hierarchical-pore MOFs by doping with other metal ions.

## Introduction

Volatile organic compounds (VOCs) as hazardous air pollutants are the precursors of ozone and smog (Zhang et al., 2019). They are derived from various industrial processes and applications of chemical products. The emissions of VOCs have caused severe environmental and healthy issues causing cancer and death (Zhu et al., 2016, 2019; Meng et al., 2019). Therefore, the reduction of VOCs emissions is of great significance. Adsorption with appropriate materials as VOC adsorbent has been known as an energy-efficient and promising strategy (Vellingiri et al., 2017; Zhang et al., 2017; Sui et al., 2019).

Metal-organic frameworks (MOFs), as novel adsorbents, have been attracted considerable attention owing to their excellent specific surface and porosity, as well as tunable topologies (Sun et al., 2014; Ahmed and Jhung, 2016; Li et al., 2018; Lin et al., 2018). Importantly, MOFs have been developed as attractive candidates for VOCs adsorption (Duan et al., 2013; Vellingiri et al., 2016, 2017; Wang et al., 2018). Hu et al. (2018) reported that UiO-66 had the high adsorption capacities of chlorobenzene (4.94 mmol/g) and acetaldehyde (9.42 mmol/g). Xian et al. (2015) indicated that the uptakes of 1,2-dichloroethane and ethyl acetate (EA) on MIL-101(Cr) were separately 9.71 and 5.79 mmol/g. Previously, our results also implied that: (1) MIL-101(Cr) exhibited excellent uptakes of a series of aromatics and *n*-alkanes (7.2–16.3 mmol/g) (Sun et al., 2014, 2017); and (2) MOF-5 possessed high uptakes of linear alkanes *n*C_4_-*n*C_7_ (8.0–11.5 mmol/g) (Lv et al., 2017). However, most MOFs (e.g., UiO-66, HKUST-1, MOF-5) are microporous with pore sizes <20 Å, which does not benefit molecule diffusion and mass transport (Huang et al., 2015; Yang et al., 2018), inhibiting their applications in VOCs adsorption separation.

Recently, hierarchical-pore MOFs (H-MOFs) are attracting wide attention because of their exceptional properties, including that the micropores can facilitate high surface area, and the mesopores or macropores can enhance molecule diffusion and mass transport (Duan et al., 2018a). Various strategies have been developed to prepare hierarchical porous MOFs, including ligand extension method, mixed ligands method, post-modification method, and template method (Bradshaw et al., 2014; He et al., 2016; Yuan et al., 2017; Chen et al., 2019). Whereas, most of these methods need rigorous experimental conditions and complicated post-treatments (Yang et al., 2018). Moreover, these strategies are disrupted by other disadvantages, such as the high cost of larger ligands, the random copolymerization of mixed ligands, the interpenetrated structures, the collapse of framework after template removal and so on (Yuan et al., 2017; Duan et al., 2018b). Therefore, it is imperative to develop a new strategy to synthesize H-MOFs with high VOCs adsorption capacities.

Notably, HKUST-1 (Cu-BTC) is a potential adsorbent for VOCs due to its high porosity, accessible unsaturated metallic sites, good thermal stability, and easy production (Sun et al., 2015; Wu et al., 2015; Vellingiri et al., 2016). HKUST-1 contains 3D intersectional pores with a window size of 6.0 Å and cage size of 9.0 Å (Zhao et al., 2015). These micropores can offer strong adsorption affinity toward VOCs molecules, but limit their diffusion rate, especially for large ones. Recently, Duan et al. prepared hierarchical-pore HKUST-1 by employing organic amines or dual-functional surfactant as template and investigated its catalytic activity (Duan et al., 2018b,c). Nevertheless, the hierarchical-pore HKUST-1 exhibited lower BET surface area (584–1,241 m^2^/g) compared to the parent HKUST-1(1,425 m^2^/g). Mao et al. (2015) developed a ligand-assisted etching process to prepare the hierarchical porous HKUST-1 with BET surface area of 1,462 m^2^/g, which showed the enhanced CO_2_ adsorption capacity and improved diffusion rate. Chuah et al. (2017) synthesized hierarchically structured HKUST-1 nanocrystals with BET surface area of 1,328 m^2^/g to improve the SF_6_ uptake and adsorption kinetics. However, these approaches are still complicated and BET surface areas of the hierarchical-pore HKUST-1 need to be further improved. Thus, it is imperative to develop a facile approach to synthesize hierarchical-pore HKUST-1 with high BET surface area and enhanced VOCs adsorption capacity.

In this work, we initiated a simple and effective method to synthesize hierarchical-pore HKUST-1 by introducing Fe^3+^ ions in the HKUST-1 synthesis process. The obtained hierarchical-pore products were characterized, and subsequently their benzene adsorption isotherms and kinetics were measured. Consecutive adsorption–desorption cycles were performed to evaluate the reversibility of benzene adsorption. Importantly, hierarchical-pore HKUST-1 exhibited high porosities with micropores and mesopores. In a result, the hierarchical-pore HKUST-1 showed outstanding benzene adsorption capacities, enhanced diffusion rate and excellent reversibility. Current work indicated that the hierarchical-pore HKUST-1 might be an attractive candidate for VOCs adsorption applications.

## Experimental

### Materials

Copper nitrate trihydrate [Cu(NO_3_)_2_·3H_2_O, 99%], ferric trichloride hexahydrate (FeCl_3_·6H_2_O, 99%), and N,N-dimethylformamide (DMF, 99.5%) were purchased from Shanghai Macklin Biochemical Co., Ltd. Benzenetricarboxylic acid (H_3_BTC, 99%) was provided from Beijing J&K Chemical Technology Co., Ltd.

### Synthesis of Fe-Doped HKUST-1

HKUST-1 was prepared according to the previous report with some modifications (Duan et al., 2018b). In general, copper nitrate trihydrate (1.45 g, 6 mmol) and H_3_BTC (0.84 g, 4 mmol) were dissolved in 36 mL DMF. The mixture was then transferred to a Teflon-lined autoclave and heated at 383 K for 21 h. The products were filtered and sequently washed with DMF and ethanol. Fe-doped HKUST-1 (Fe-HK) samples with different Fe^3+^/Cu^2+^ molar ratios were synthesized following the similar procedure of HKUST-1, in which the total mole value (6 mmol) of Fe^3+^ and Cu^2+^ was constant by substitution of copper nitrate trihydrate with ferric trichloride hexahydrate. Herein, Fe-HK samples were synthesized with initial Fe^3+^/Cu^2+^ molar ratios of 0.15, 0.20, 0.33, and 0.50, and labeled as Fe-HK-1, Fe-HK-2, Fe-HK-3, and Fe-HK-4, respectively.

## Results and Discussion

### Physical Characteristics

**Figure 1:**
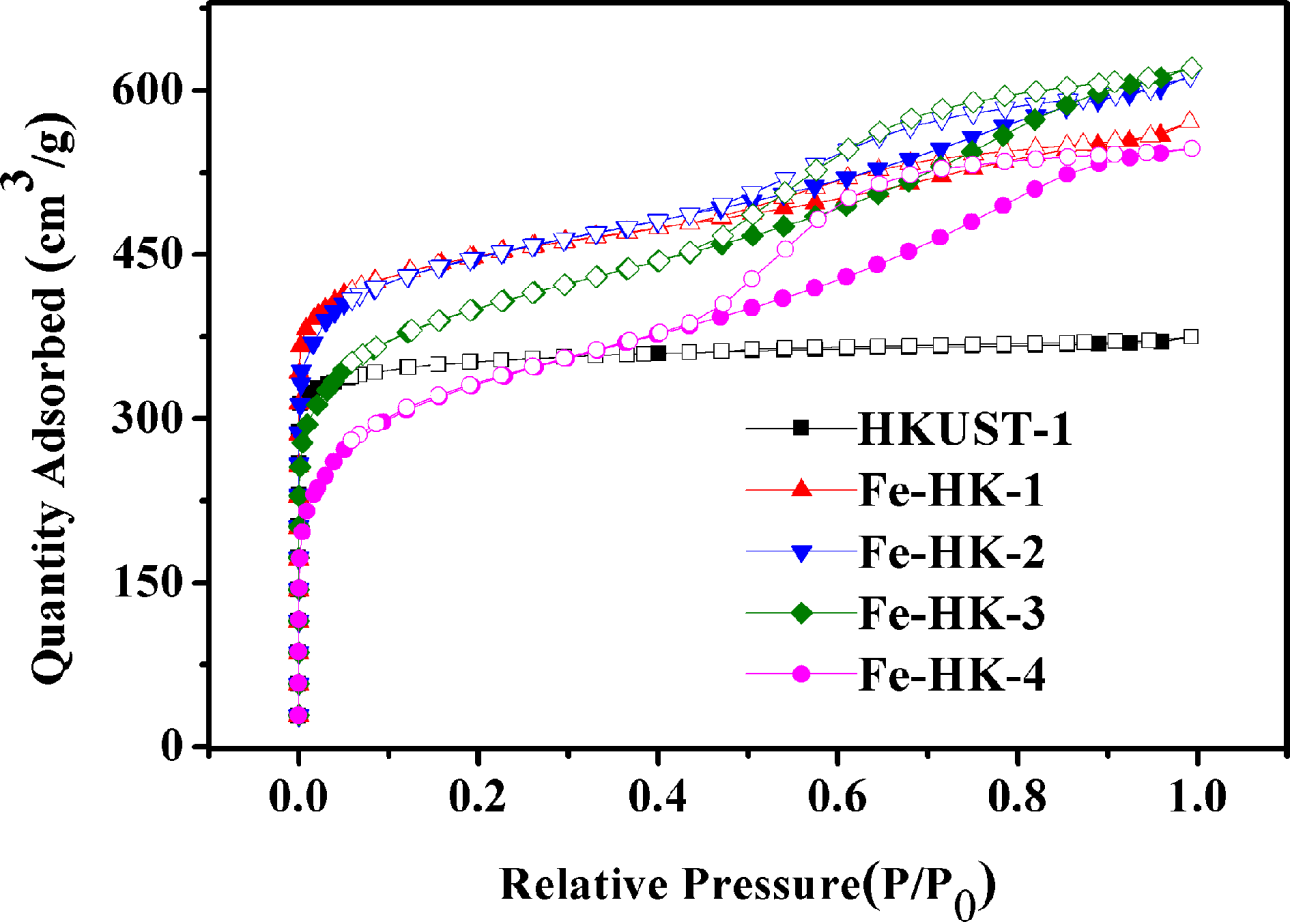
N_2_ isotherms of HKUST-1 and Fe-HK series samples at 77 K.

**Figure 2:**
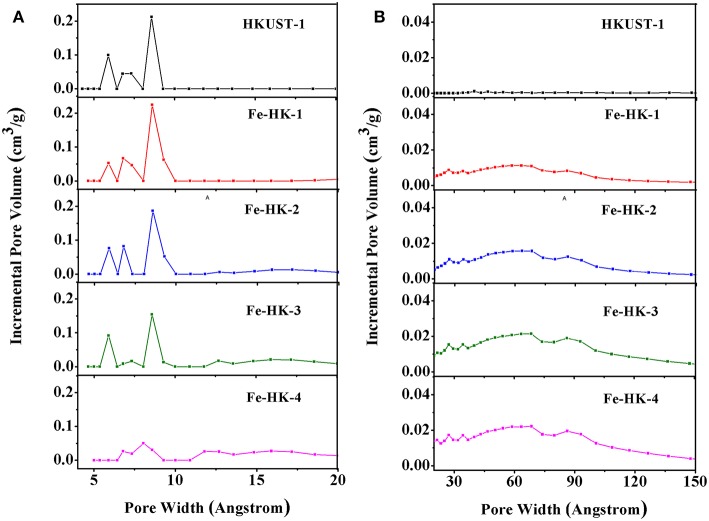
Pore size distribution curves of HKUST-1 and Fe-HK samples **(A)** micropore and **(B)** mesopore.

Figure 1 shows the *N*_2_ isotherms of HKUST-1 and four Fe-HK samples at 77 K. The isotherm of HKUST-1 exhibits the typical type-I characteristic, indicating the existence of micropores. Whereas, all the Fe-HK samples exhibit typical type-IV isotherms with apparent hysteresis loops, implying the presence of mesopores. These results are further confirmed by NLDFT pore-size distribution curves of these materials. As shown in Figure 2, HKUST-1 only presents micropores below 10 Å. The pore size below 10 Å is also observed in all Fe-HK samples, suggesting that the porous structure of HKUST-1 is remained in the Fe-HK samples. Comparing to HKUST-1, there are also new micropores (10–20 Å) and mesopores in Fe-HK samples. On one hand, the coordination between Fe^3+^ and carboxylic groups in the BTC can form new micropores and smaller mesopores. On the other hand, Fe^3+^ ions can coordinate with BTC ligand and compete with Cu^2+^ ions to result in the formation of defects and larger mesopores (Horcajada et al., 2014; Zhou et al., 2016). Moreover, the volumes of mesopores increase with Fe^3+^ contents in the Fe-HK samples. These results indicate that Fe-HK samples exhibit hierarchical porous structures.

Table 1 lists the textural properties of HKUST-1 and the Fe-HK samples. Fe-HK-1-3 samples possess higher BET surface areas, micropore volumes and mesopore volumes than HKUST-1, suggesting that the small addition of Fe^3+^ can enhance the porosities of Fe-HK samples. This may be ascribed to the formation of new microporous and mesoporous structures owing to the introduction of Fe^3+^ (Horcajada et al., 2014; Zhou et al., 2016). Additionally, with the increase of molar ratio of Fe^3+^/Cu^2+^ from 0.15 to 0.33, the BET surface areas and micropore volumes of the three Fe-HK samples (Fe-HK-1-3) gradually decrease from 1,729 to 1,495 m^2^/g and 0.64–0.53 cm^3^/g, respectively. In contrast, the total pore volumes, mesopore volumes of the three samples increase with the molar ratio of Fe^3+^/Cu^2+^, and could separately reach 0.95 and 0.42 cm^3^/g. However, Fe-HK-4 (Fe^3+^/Cu^2+^ = 0.5) shows lower BET surface area than HKUST-1, which is possibly attributed to the formation of the serious crystal defects owing to the excess Fe^3+^ (Zhou et al., 2016). After comprehensive consideration, Fe-HK-2 (Fe^3+^/Cu^2+^ = 0.20) was chosen as the optimal sample, which presents BET surface area of 1,707 m^2^/g and total pore volume of 0.93 cm^3^/g, higher than the previously reported values for hierarchical-pore HKUST-1 prepared with different methods (Mao et al., 2015; Duan et al., 2018b,c).

**Table 1:** Pore structure parameters of HKUST-1 and Fe-HK samples.

**Sample**	**Surface Area (m^**2**^/g)**	**Pore Volume (cm**^****3****^**/g)**		
	**BET**	**Total**	**Micropore**	**Mesopore**
HKUST-1	1400	0.56	0.48	0.08
Fe-HK-1	1729	0.86	0.64	0.22
Fe-HK-2	1707	0.93	0.62	0.31
Fe-HK-3	1495	0.95	0.53	0.42
Fe-HK-4	1226	0.84	0.41	0.43

**Figure 3:**
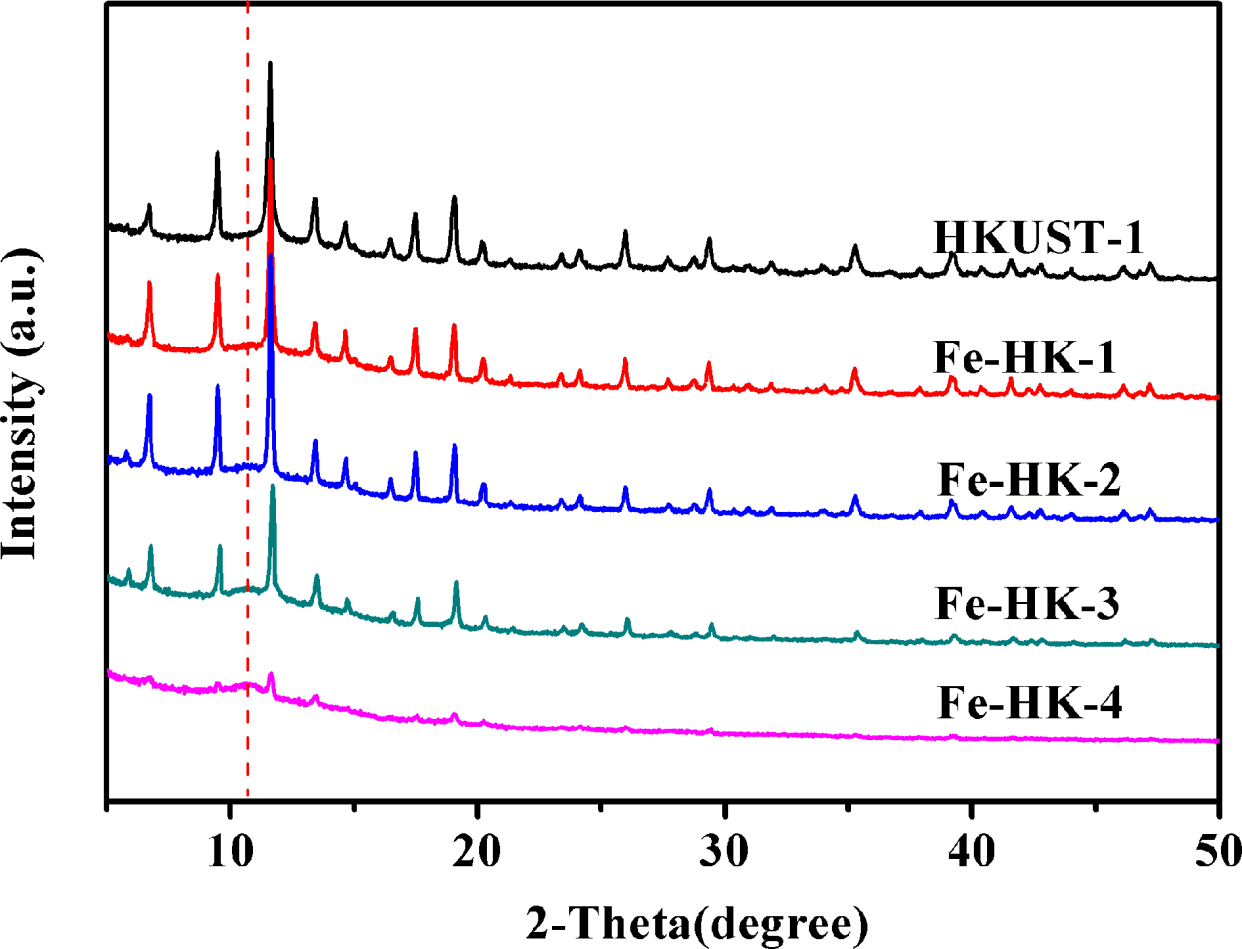
PXRD patterns of HKUST-1 and the Fe-HK samples.

Figure 3 shows the PXRD patterns of HKUST-1 and the Fe-HK samples. The PXRD patterns of Fe-HK-1-3 samples exhibit the main characteristic peaks at 9.4°, 11.6°, 17.5°, and 19.1°, which are identical to that of HKUST-1. It indicates that the Fe-HK-1-3 samples preserve the crystalline characters of HKUST-1, whereas, Fe-HK-4 shows very weak characteristic peaks of HKUST-1 and a new characteristic peak at 10.7° appears. It suggests that when the Fe^3+^ content is higher than certain number, the growth of the HKUST-1 structure would be prevented (Ebrahim and Bandosz, 2013; Zhou et al., 2016). This conclusion is in agreement with the above BET results. Additionally, the new peak at 10.7° may be due to the interaction between Fe^3+^ and carboxylic groups in the BTC.

**Figure 4:**
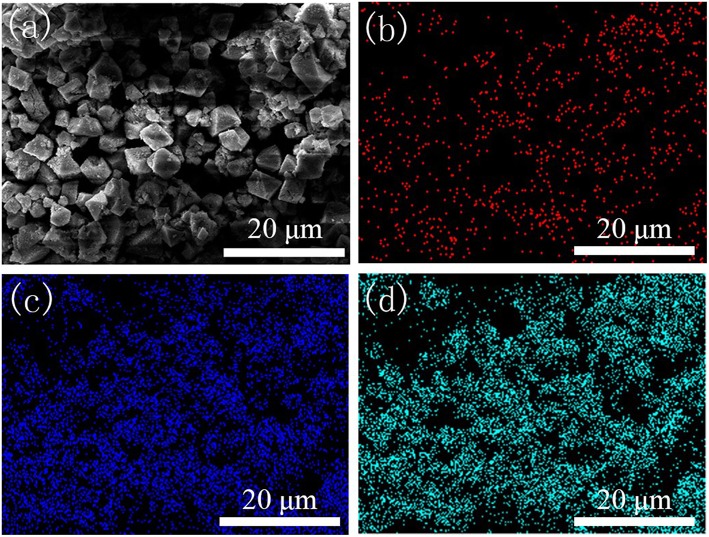
EDS mapping of Fe-HK-2 sample. SEM image **(a)** and the corresponding elemental distributions of Fe **(b)**, Cu **(c)**, and O **(d)**.

SEM images of HKUST-1 and the Fe-HK samples are presented in Figure S1. It indicates that HKUST-1 has octahedral morphology. The Fe-HK-1-3 samples maintain octahedral structure and their surfaces become rough due to the incorporation of Fe^3+^. Similar results were also found for other metal ion doping MOFs (Ebrahim and Bandosz, 2013). EDS mapping of the Fe-HK-2 sample was selected as a representative to verify the existence and dispersion of iron ions. As shown in Figure 4, the existence of Fe element is clearly confirmed. It is also observed that the Fe and Cu elements are well-dispersed in the Fe-HK-2 crystals, indicating that the iron ions were incorporated successfully into the HKUST-1 framework. The EDS results of Fe/Cu atomic ratios for all Fe-HK samples are listed in Table S1. It indicates that the atomic ratios of Fe/Cu in Fe-HK-1, Fe-HK-2, Fe-HK-3 and Fe-HK-4 are 0.17, 0.21, 0.31, and 0.53, respectively, which are very close to their design values.

**Figure 5:**
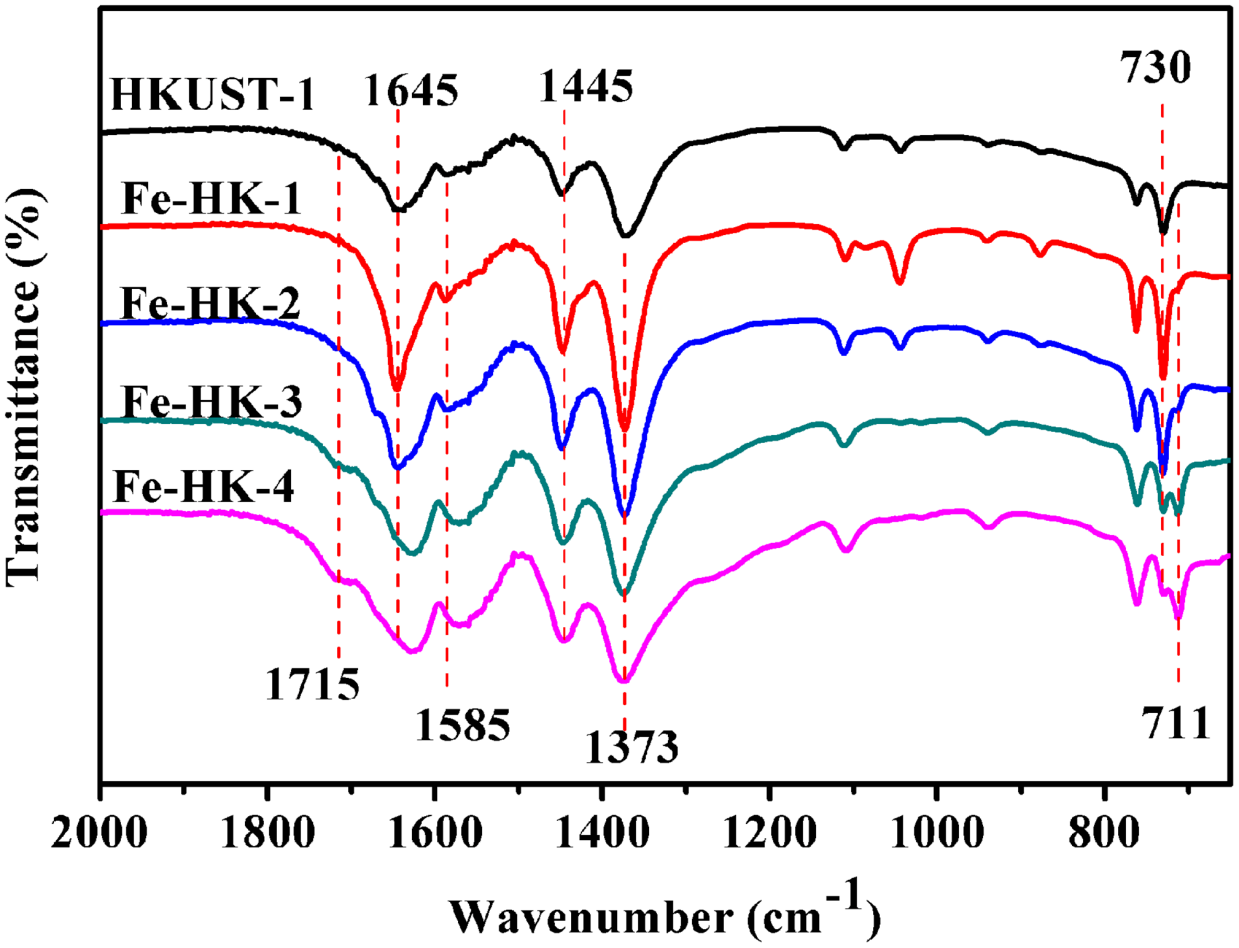
FTIR spectra of HKUST-1 and Fe-HK samples.

Figure 5 displays FTIR spectra of HKUST-1 and the Fe-HK samples. These FTIR spectra are nearly similar, which present the asymmetric vibrations of carboxylate groups at 1,645/1,585 cm^−1^ and the symmetric vibrations at 1,445/1,373 cm^−1^ (Petit et al., 2012; Sun et al., 2015). However, it is noticed that for Fe-HK-3 and Fe-HK-4, the two bands at 1,645/1,585 cm^−1^ shift to lower wavenumbers, and a new band appears at 1,715 cm^−1^ belonged to the partially coordinated trimesic acid (Majano et al., 2014). Moreover, the band at 730 cm^−1^ is ascribed to the vibration of Cu-O, in which copper ions coordinate with the carboxylate groups of BTC (Lin et al., 2014). A new band appears at 711 cm^−1^, which is ascribed to the vibration of Fe-O, indicating that iron ions coordinate with the carboxylate groups of BTC (Tan et al., 2015; Abdpour et al., 2018). The peak intensity at 711 cm^−1^ becomes stronger, while the peak intensity at 730 cm^−1^ becomes weaker as the increase of molar ratio of Fe/Cu.

**Figure 6:**
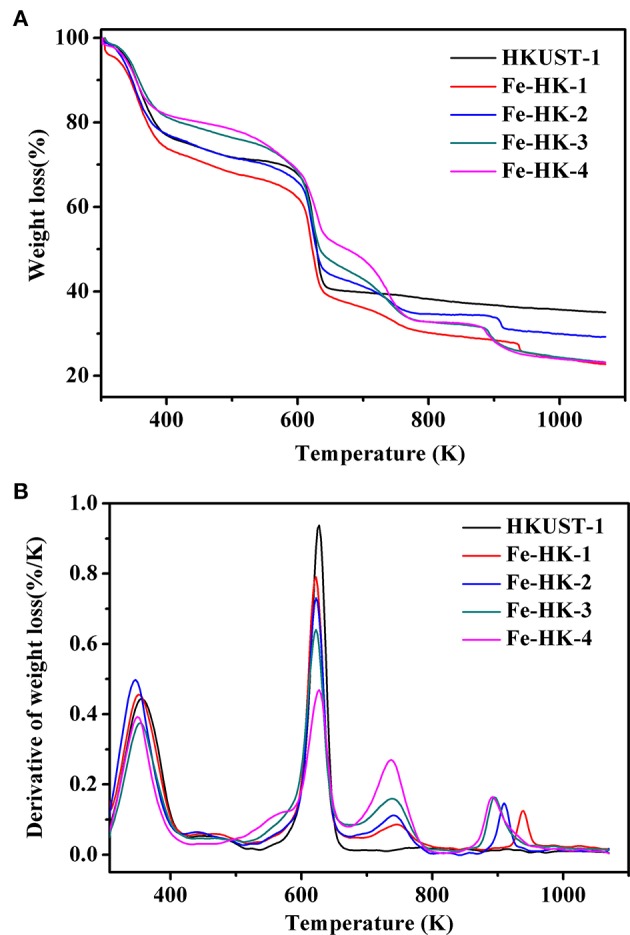
TG **(A)** and DTG **(B)** curves of HKUST-1 and Fe-HK samples.

Figure 6 exhibits TG and DTG curves of HKUST-1 and the Fe-HK samples. It is apparent that HKUST-1 and the Fe-HK samples present the different weight loss patterns. HKUST-1 exhibits two weight loss steps. The first weight loss below 373 K corresponds to the loss of guest molecules. The second weight loss between 523 and 673 K is assigned to the removal of BTC ligands coordinated to Cu^2+^ and the formation of CuO (Lin et al., 2012). Interestingly, all the Fe-HK samples exhibit four weight loss steps in Figure 6B. The first peak is similar to that of HKUST-1. The second peak broadens after the incorporation of Fe^3+^ ions. Thus, the second weight loss of the Fe-HK samples can correspond to the decomposition of organic ligands linked to Cu^2+^ and Fe^3+^ ions to form CuO and Fe_3_O_4_ at the same time (Petit and Bandosz, 2012). Additionally, a new peak as the third weight loss reveals between 673 and 800 K, which is ascribed to the reduction of Fe_3_O_4_-Fe_2_O_3_ (Petit and Bandosz, 2012). Another new peak as the fourth weight loss occurs between 800 and 1,000 K, which is due to the reduction of Fe_2_O_3_ to FeO (Petit and Bandosz, 2012).

### Adsorption Isotherms of Benzene

**Figure 7:**
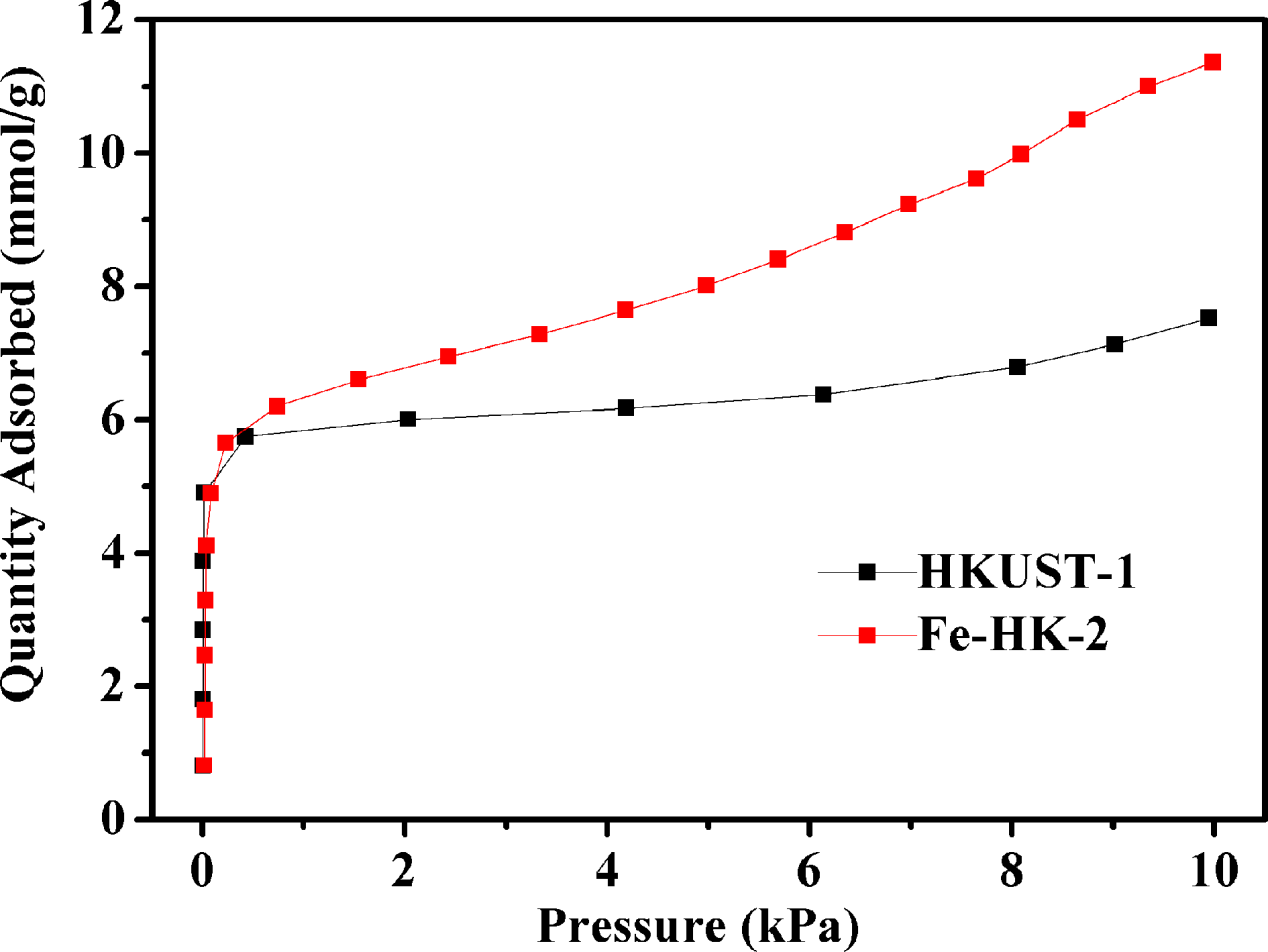
Adsorption isotherms of benzene on HKUST-1 and Fe-HK-2 at 298 K.

Figure 7 presents adsorption isotherms of benzene on HKUST-1 and the optimal sample Fe-HK-2 at 298 K. It is observed that benzene adsorption capacities of HKUST-1 and Fe-HK-2 exhibit a sharp rise at low pressure (<0.5 kPa), which is ascribed to the micropore adsorption owing to the metal –π interactions between benzene molecules and the metal cations as well as the π-π stacking and electrostatic interactions between benzene molecules and the organic ligand (Sun et al., 2017). Subsequently, benzene adsorption capacity of HKUST-1 reaches a plateau followed by a slight increase at 8 kPa, which is related to the intermolecular π-π interactions of benzene (Zhu et al., 2017). Nevertheless, benzene adsorption capacity of Fe-HK-2 presents a gradual increase at high pressure, which is mainly attributed to the mesopore filling due to the π-π and electrostatic interactions between benzene molecules and the organic ligand as well as the intermolecular benzene–benzene interactions (Sun et al., 2017). Moreover, the Fe-HK sample exhibits higher saturated adsorption capacity of benzene than HKUST-1. The maximum adsorption capacity of benzene on Fe-HK-2 is up to 11.4 mmol/g, which is 1.5 times higher than that on HKUST-1(7.5 mmol/g). The great increase in benzene capacity of Fe-HK-2 can be attributed to its higher BET surface area and pore volume than HKUST-1.

**Figure 8:**
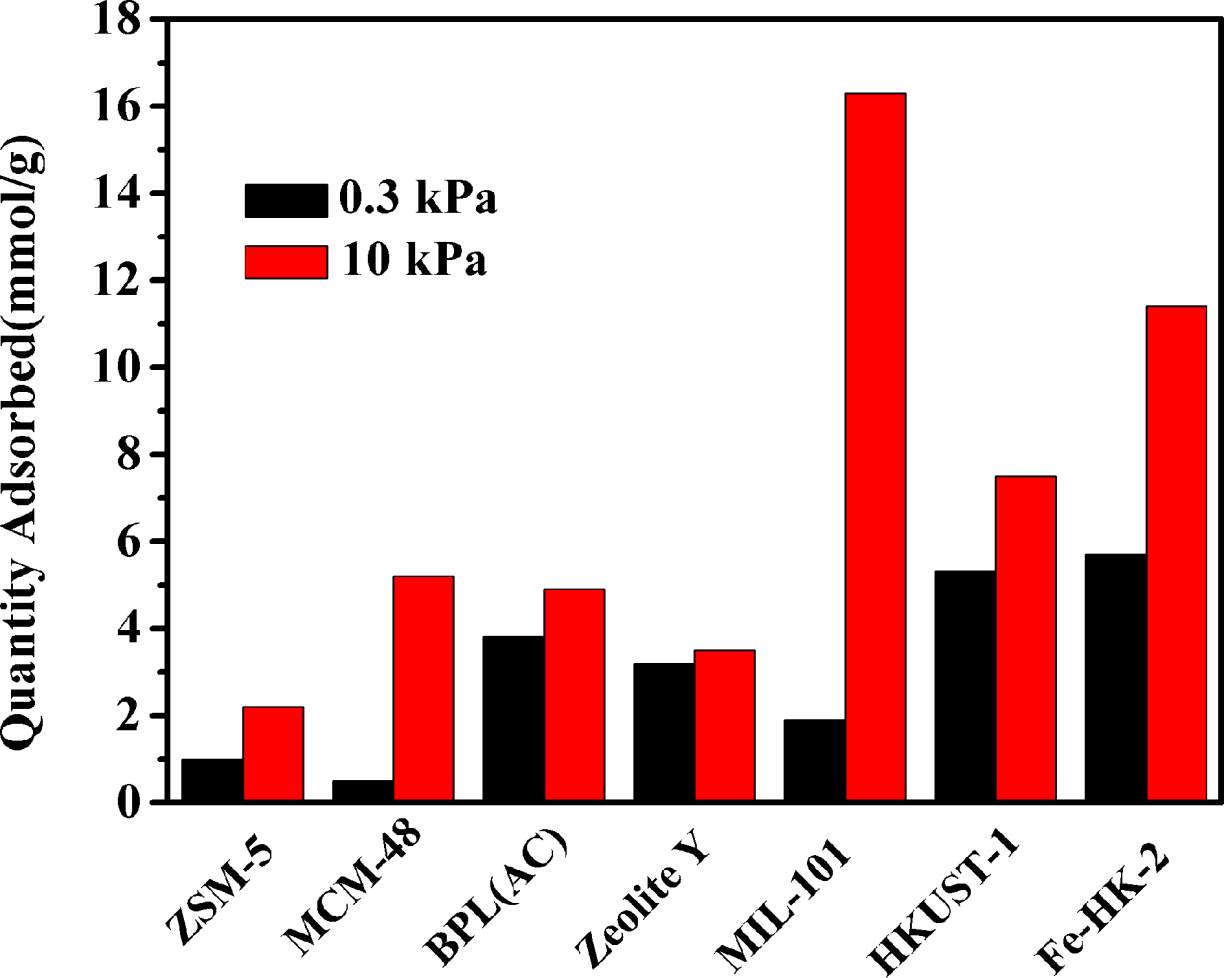
Adsorption capacity comparison of benzene on some adsorbents at same conditions.

For comparison, the adsorption capacities of benzene on some typical porous materials at 0.3 and 10 kPa are shown in Figure 8. It indicates that the synthesized HKUST-1 and Fe-HK-2 possess much higher benzene capacities than the conventional adsorbents, e.g., ZSM-5 (Jhung et al., 2007), MCM-48 (Hartmann and Bischof, 1999), BPL(AC) (Zhao et al., 1998) and Zeolite Y (Zhao et al., 1998). It is also seen that the uptake of benzene on Fe-HK-2 at high pressure 10 kPa is lower than some MOFs [i.e., MIL-101 (Sun et al., 2017)], whereas, the benzene adsorption capacity of 5.7 mmol/g on the Fe-HK-2 at low pressure 0.3 kPa is 3 times larger than that of MIL-101(1.9 mmol/g). One reason might be that Fe-HK-2 with smaller micropore size is favored for benzene adsorption at low pressure in comparison with MIL-101. Therefore, the hierarchical Fe-HK-2 exhibits certain advantages over some conventional adsorbents or MOFs.

### Adsorption Kinetics of Benzene

**Figure 9:**
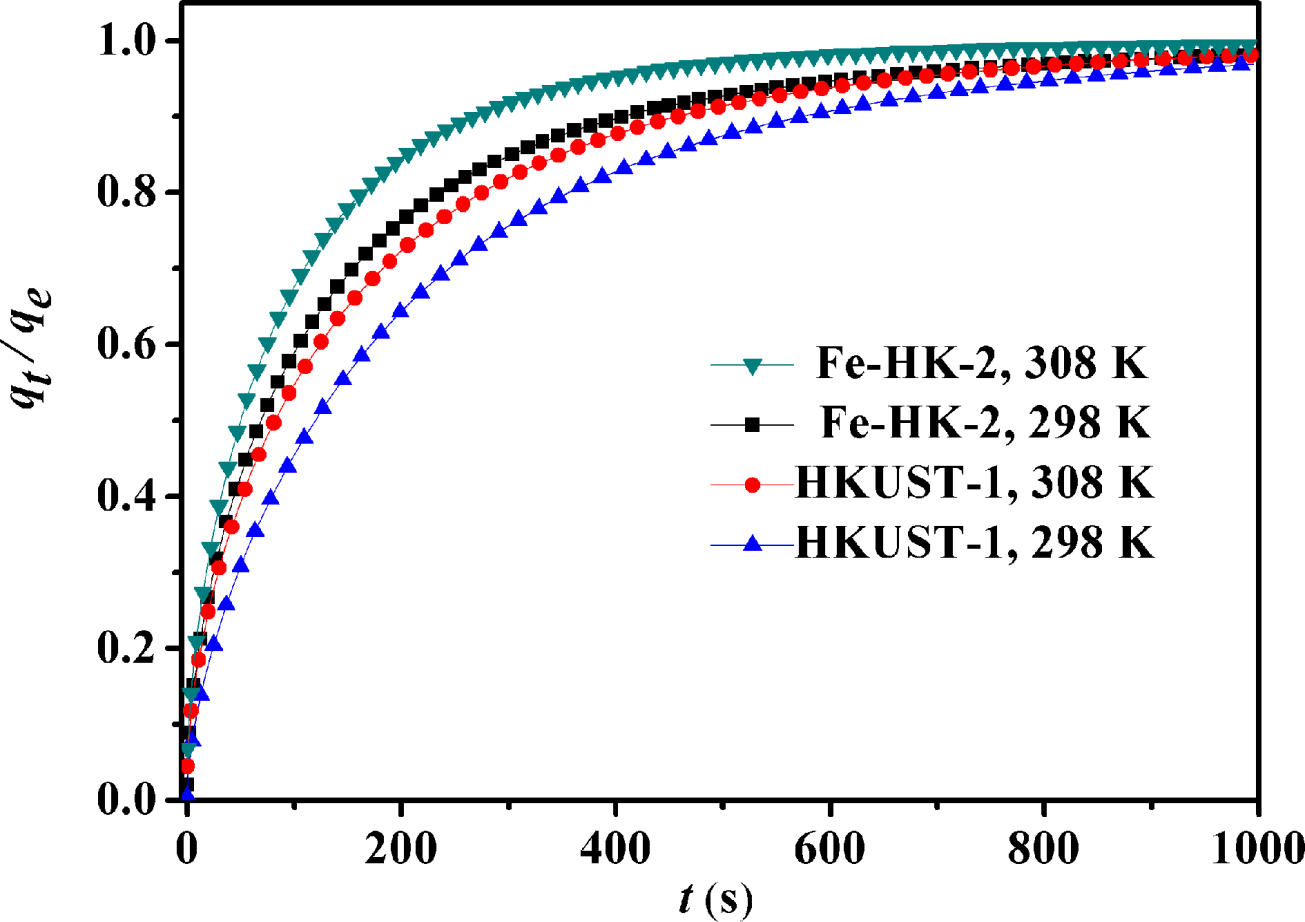
Fractional uptakes of benzene on HKUST-1 and Fe-HK-2 at 0.2 kPa.

**Figure 10:**
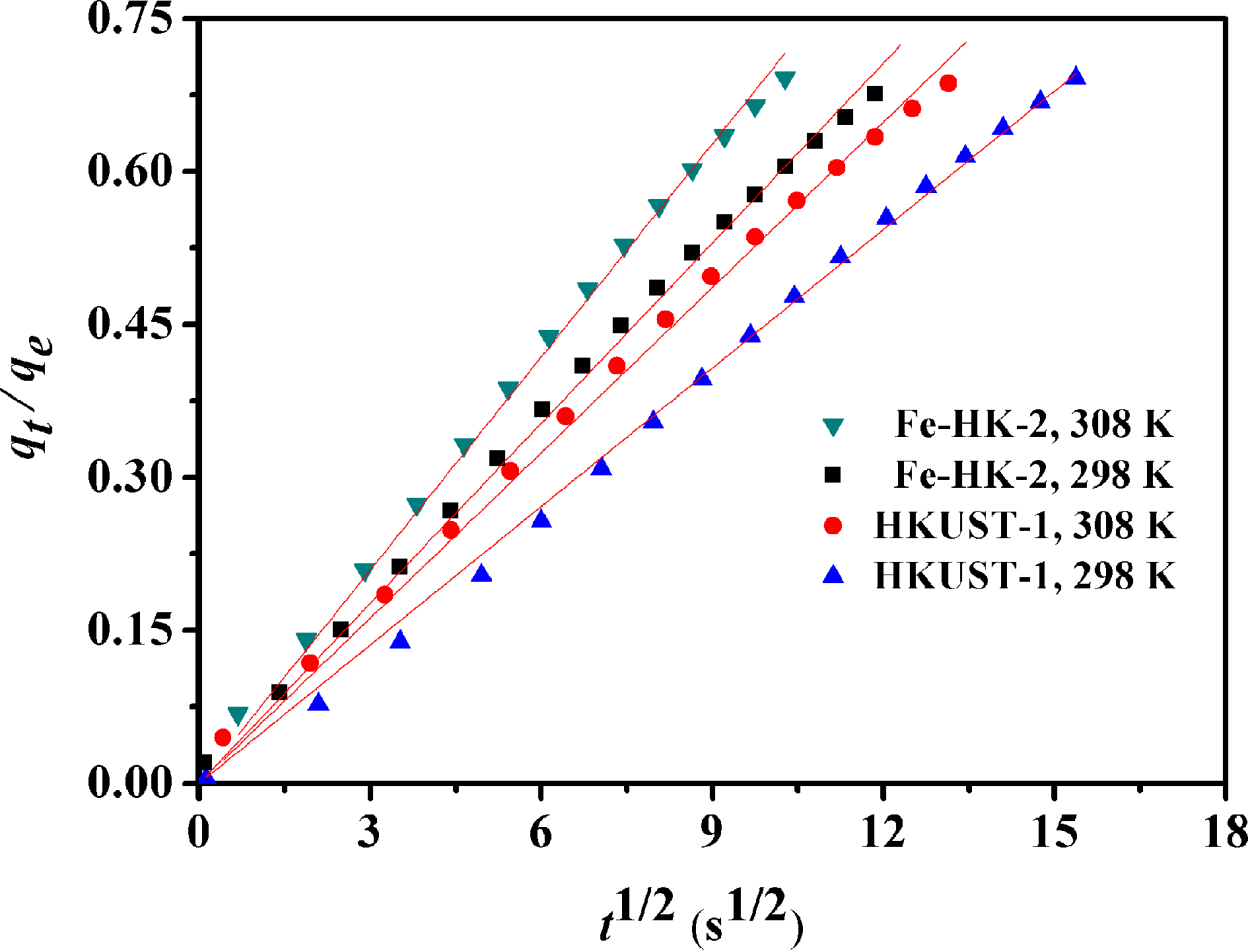
Plots of the fractional benzene uptakes (*q*_*t*_/*q*_e_) against the square root of adsorption time at 298 and 308 K.

Figure 9 shows the kinetic curves of benzene on HKUST-1 and Fe-HK-2 at 298 and 308 K. Compared to HKUST-1, equilibrium uptakes of benzene on Fe-HK-2 are achieved in a shorter time owing to the presence of mesopores. In order to obtain the intracrystalline diffusion coefficient of benzene on HKUST-1 and Fe-HK-2, the plots of fractional uptake (*q*_*t*_/*q*_e_) of benzene vs. the square root of adsorption time at 298 and 308 K are presented in Figure 10. The diffusion time constants (*D*_M_/$r_{\text{c}}^{2}$, s^−1^) were obtained from the slope of *q*_*t*_/*q*_e_ vs. $\sqrt{t}$ based on the diffusion model (Equation 1) (Sui et al., 2013; Zhu et al., 2017). Their activation energy (*E*_a_) was estimated by Arrhenius equation (Equation 2) (Hu et al., 2018).

(1) $$\begin{array}{r} \frac{q_{t}}{q_{e}}≅\frac{6}{r_{c}}\sqrt{\frac{D_{M}t}{\pi }} \end{array}$$

(2) $$\begin{array}{r} 1n{\;D}_{M}=1n\;A-\frac{E_{a}}{RT} \end{array}$$

where *q*_e_ (mg/g) and *q*_*t*_ (mg/g) are the uptakes of benzene on adsorbent per gram at equilibrium and time *t, D*_M_ (cm^2^/s) is the intracrystalline diffusion coefficient, *r*_c_ (cm) is the crystal radius, R is the gas constant and A is the Arrhenius factor.

Table S2 lists benzene diffusivity parameters and the activation energies of HKUST-1 and Fe-HK-2. The high regression coefficients (*R*^2^ >0.996) suggests that the diffusion model can fit the kinetic curves of benzene well. The diffusion time constants (*D*_M_/$r_{\text{c}}^{2}$) of benzene on the HKUST-1 and Fe-HK-2 are in the range of (1.78–2.54) × 10^−4^ and (3.02–4.23) × 10^−4^ s^−1^, respectively. Corresponding diffusion coefficients (*D*_M_) using the average particle radius *r*_c_ of 4 μm (Figure S1) are (2.85–4.07) × 10^−11^ and (4.83–6.76) × 10^−11^ cm^2^/s for HKUST-1 and Fe-HK-2, respectively. The diffusivity value of Fe-HK-2 is 1.7 times faster than that of parent HKUST-1. It is also noticed from Table S2 that the activation energy *E*_a_ for diffusion of benzene on Fe-HK-2 is about 25.78 kJ/mol, which is smaller than that on HKUST-1 owing to faster benzene diffusion on Fe-HK-2. These results indicate that the Fe-HK-2 with mesopores exhibits the enhanced diffusion and mass transport than HKUST-1.

### Recycling Performance of Fe-HK-2 for Benzene Adsorption

**Figure 11:**
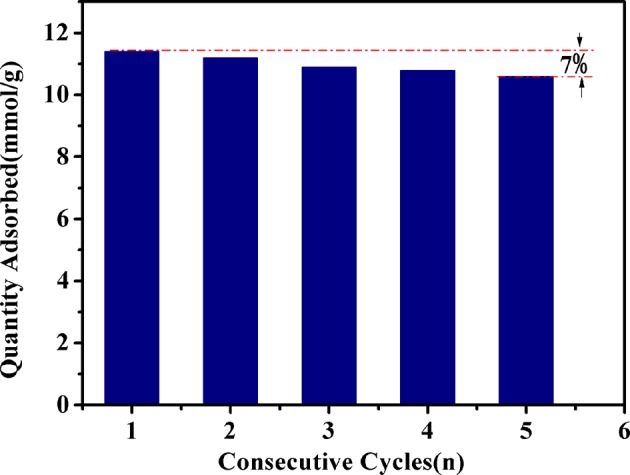
Adsorption capacities of benzene after consecutive cycles on Fe-HK-2 at 298 K.

The reversibility of Fe-HK-2 for benzene adsorption was also tested in this work. Adsorption was measured at 10 kPa, then desorption was performed at 373 K under high vacuum. Figure 11 presents adsorption capacities of benzene after consecutive cycles on Fe-HK-2 at 298 K. After five consecutive cycles, the adsorption capacities of benzene on Fe-HK-2 decreases by only 7%, indicating that the Fe-HK-2 possesses an outstanding reversibility of VOCs adsorption.

## Conclusions

In summary, we proposed a facile strategy to prepare novel hierarchical Fe(III)-doped Cu-MOF materials with enhanced benzene adsorption. The characterizations showed that the introduction of Fe^3+^ ions could form new mesopores in Fe-HK. The hierarchical-pore Fe-HK-2 exhibited high BET surface area of 1,707 m^2^/g, mesopore volume of 0.31 cm^3^/g and total pore volume of 0.93 cm^3^/g, which are much higher than those of the original HKUST-1. This improves benzene adsorption capacity of Fe-HK-2, showing 1.5 times greater than that on HKUST-1. Fe-HK-2 also exhibited faster diffusivity and excellent reversibility of benzene adsorption. Thus, the hierarchical-pore Fe-HK-2 could be a promising adsorbent for VOCs adsorption. The results also indicate that metal ion doping can be a facile strategy to synthesize hierarchical-pore MOFs with enhanced adsorption performance.

## Data Availability Statement

The datasets generated for this study are available on request to the corresponding author.

## Author Contributions

XS, XG, WX, and W-JC have provided substantial contribution to the acquisition, interpretation and analysis of data, as well as to the drafting of the work. QX, XP, XZ, YL, and Q-HW performed essential work regarding to the conception and design of the research presented and to its critical revision for important intellectual content. They all approve the version to be published and agree to be accountable to investigate and resolve any question related to the accuracy or integrity of any part of the work.

### Conflict of Interest

The authors declare that the research was conducted in the absence of any commercial or financial relationships that could be construed as a potential conflict of interest.
