# Supplementary material for: Novel Hierarchical Fe(III)-Doped Cu-MOFs With Enhanced Adsorption of Benzene Vapor
Source: Front Chem. 2019 Sep 27;7:652. doi: 10.3389/fchem.2019.00652 (PMC6776884; doi:10.3389/fchem.2019.00652)
Supplement: Supplementary file 1 [file Data_Sheet_1.docx]

Supplementary Material

**Novel Hierarchical Fe(Ⅲ)-Doped Cu-MOFs with Enhanced**

**Adsorption of Benzene Vapor**

**Xuejiao Sun^1^, Xiulian Gu^1^, Wentao Xu^1^, Wen-Jie Chen^1^, Qibin Xia^2*^, Xiaoyang Pan^1^, Xiaojing Zhao^1^, Yi Li^3^, Qi-Hui Wu****^4*^**

^1^ School of Chemical Engineering and Materials Science, Quanzhou Normal University, Quanzhou, China

^2^ School of Chemistry and Chemical Engineering, South China University of Technology, Guangzhou, China

^3^ Jiangsu Key Laboratory of Advanced Functional Polymer Design and Application, Department of Polymer Science and Engineering, College of Chemistry, Chemical Engineering and Materials Science, Soochow University, Suzhou, China

^4^ College of Mechanical and Energy Engineering, Jimei University, Xiamen, China

*** Correspondence:**Corresponding Author
qbxia@scut.edu.cn (Qibin Xia)

qihui_wu@xmu.edu.cn (Qi-Hui Wu)

# Characterization

Powder X-ray diffraction (PXRD) data were obtained from a Bruker D8 Advance X-ray diffractometer using Cu Kα radiation with a scan rate of 2º/min and a step size of 0.02^o^ in 2θ. Scanning electron microscope (SEM) was conducted on a ZEISS MERLIN Compact. Scanning was carried out on the samples previously dried and sputter-coated with a thin layer of gold. The FTIR spectra were collected on a Bruker Vector 33 spectrometer. Thermogravimetric analysis (TGA) was performed on a TA Q500 instrument heating from 303 to1073 K in nitrogen atmosphere at a rate of 10 K/min. N_2_ isotherms of the samples were obtained at 77 K on 3Flex Surface Characterization Analyzer (Micromeritics Instrument Corporation, USA) equipped with the commercial calculation and analysis software. The pore textural properties including BET surface area and pore volume were determined based on the N_2_ adsorption/desorption isotherm. Pore size distribution calculation was provided by 3Flex equipped with the software based on non-local density functional theory (NLDFT).

# Adsorption measurements

The adsorption isotherms of benzene vapor were measured at 298 K using the Micromeritics 3Flex Surface Characterization Analyzer. The vapor generation system included a stainless steel chamber with a hard seal, manual cutoff valve to be attached in place of the Psat tube, and a heating mantle to control the temperature of the chamber at an operator-specified temperature between ambient and 316 K. The constant adsorption temperature was achieved by putting sample cell into circulating water bath. Before the adsorption measurements, the vapor vessel was filled with the benzene, and then kept at 313K to generate the vapor. Each sample was outgassed at 423 K for 8 h under vacuum prior to the measurements.

The adsorption kinetic curves of benzene were explored by using the intelligent gravimetric analyzer (IGA-003, Hiden). Each sample (40–50 mg/run) was degassed at 423 K for 6 h under vacuum. The measurements were performed at 298-308 K and 0.2 kPa. The adsorption capacities of benzene on the sample can be presented as

$q_{e}=\frac{1000(W_{e}-W_{a})}{W_{a}}$ (1)

$q_{t}=\frac{1000(W_{t}-W_{a})}{W_{a}}$ (2)

where *W*_e_ (g) and *W_t_* (g) are the weights of adsorbent at equilibrium and time *t*, *W*_a_ (g) is the initial weight of adsorbent, *q*_e_ (mg/g) and *q_t_* (mg/g) are the uptakes of benzene on adsorbent per gram at equilibrium and time *t*, respectively.

# Supplementary Figures and Tables


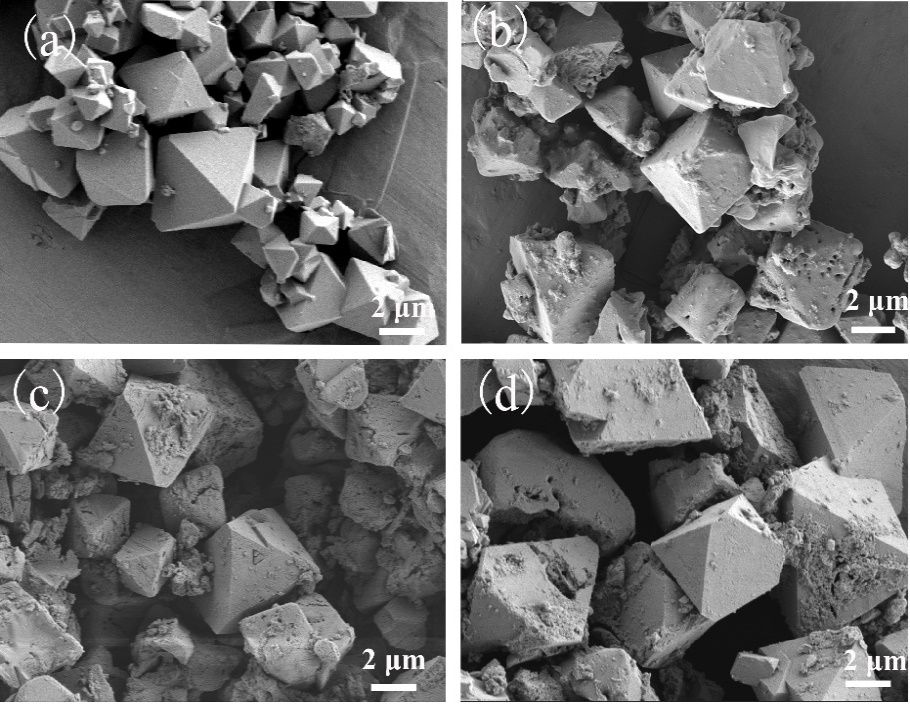


**Figure S1** SEM images of (a) HKUST-1, (b) Fe-HK-1，(c) Fe-HK-2 and (d) Fe-HK-3.

**Table S1**

EDS analysis of all Fe-HK samples.

| Sample | C  (wt%) | O  (wt%) | Fe  (wt%) | Cu  (wt%) | Fe/Cu atomic ratio |
| --- | --- | --- | --- | --- | --- |
| Fe-HK-1 | 42.18 | 25.73 | 4.09 | 28.00 | 0.17 |
| Fe-HK-2 | 40.57 | 28.37 | 4.82 | 26.24 | 0.21 |
| Fe-HK-3 | 41.02 | 28.14 | 6.57 | 24.27 | 0.31 |
| Fe-HK-4 | 43.34 | 28.46 | 9.00 | 19.20 | 0.53 |

**Table S2**

Benzene diffusivity parameters and the activation energies of HKUST-1 and Fe-HK-2.

| Sample | *D*_M_·*r*_c_^-2^ ×10^4^(s^-1^) | | *D*_M_×10^11^(cm^2^·s^-1^) | | *E*_a_  (kJ/mol) | *R*^2^ |
| --- | --- | --- | --- | --- | --- | --- |
|  | 298 K | 308 K | 298 K | 308 K |  |  |
| HKUST-1 | 1.78 | 2.54 | 2.85 | 4.07 | 27.13 | >0.996 |
| Fe-HK-2 | 3.02 | 4.23 | 4.83 | 6.76 | 25.78 | >0.996 |
